# The Mediating Role of Family and Food-Related Life Satisfaction in the Relationships between Family Support, Parent Work-Life Balance and Adolescent Life Satisfaction in Dual-Earner Families

**DOI:** 10.3390/ijerph15112549

**Published:** 2018-11-14

**Authors:** Berta Schnettler, Edgardo Miranda-Zapata, Germán Lobos, Mahia Saracostti, Marianela Denegri, María Lapo, Clementina Hueche

**Affiliations:** 1 Facultad de Ciencias Agropecuarias y Forestales, Universidad de La Frontera, Temuco 4811230, Chile; 2 Escuela de Economía, Universidad Católica de Santiago de Guayaquil, Guayaquil 090615, Ecuador; globos@utalca.cl; 3 Scientific and Technological Bioresource Nucleus (BIOREN-UFRO), Universidad de La Frontera, Temuco 4811230, Chile; 4 Núcleo Científico y Tecnológico en Ciencias Sociales, Universidad de La Frontera, Temuco 4811230, Chile; marianela.denegri@ufrontera.cl (M.D.); clementina.hueche@ufrontera.cl (C.H.); 5 Núcleo Científico y Tecnológico en Ciencias Sociales, Universidad de La Frontera, Temuco 4811230, Chile; edgardo.miranda@ufrontera.cl; 6 Facultad de Economía y Negocios, Universidad de Talca, Talca 3460000, Chile; 7 Núcleo Científico y Tecnológico en Ciencias Sociales, Universidad de La Frontera, Temuco 4811230, Chile; mahia.saracostti@ufrontera.cl; 8 Facultad de Educación, Ciencias Sociales y Humanidades, Universidad de La Frontera, Temuco 4811230, Chile; 9 Escuela de Economía, Universidad Católica de Santiago de Guayaquil, Guayaquil 090615, Ecuador; maria.lapo@cu.ucsg.edu.ec

**Keywords:** subjective well-being, adolescents, family support, work-life balance, mediating role

## Abstract

This study explored the associations between family support and satisfaction with life, food-related life and family life. It also assessed the associations between both parents’ work-life balance and satisfaction with life, food-related life and family life among adolescent children from dual-earner families. Questionnaires were administered to 303 dual-earner families with one child between 10 and 17 years in Temuco, Chile. Adolescents answered the Satisfaction with Life Scale (SWLS), Satisfaction with Food-related Life scale (SWFoL), Satisfaction with Family Life scale (SWFaL) and the Family subscale of the Multidimensional Scale of Perceived Social Support. Both parents answered the Work-life Balance (WLB) scale. Using structural equation modelling and having controlled for adolescents’ gender, age and socioeconomic status, we confirmed that adolescent life satisfaction is associated with satisfaction with family life and food-related life. Food-related life satisfaction and family life satisfaction had complete mediating roles between perceived family support and adolescents’ life satisfaction. Satisfaction with food-related life also had a complete mediating role between both parents’ WLB and adolescents’ life satisfaction. Satisfaction with family life had a complete mediating role between mothers’ WLB and adolescents’ life satisfaction. In addition, mothers’ WLB was positively associated with perceived family support among adolescents. These findings suggest the need to improve family support and work-life balance among mothers in order to enhance adolescents’ satisfaction with different domains of life in dual-earner families.

## 1. Introduction

Although female participation in the Chilean labor market is below average for Latin America (55%) and developed countries (61%), it increased from 31% in 1990 to 48% in 2017 [1], thereby changing traditional roles within the family. Though this situation provides important economic and other benefits to women and their families, it has caused changes in Chilean families, such as the sustained increase in the number of families in which both parents work [2]. Although this implies a double source of income, it also means that the time dedicated to the family decreases while pressure also increases for both parents [3,4]. However, the tension that both parents experience between parenting and work not only affects them and their relationship but can also negatively affect their relationship with their children [2,5].

During adolescence emotional and psychological well-being is easily affected by life circumstances [6,7]. Subjective well-being is an assessment people make of their own lives. This assessment includes cognitive and emotional aspects of experience. The cognitive component of subjective well-being is satisfaction with life, which is defined as a positive evaluation that a person makes of their life in general or within specific domains [8]. Bottom-up theoretical approaches to life satisfaction imply that life satisfaction depends on the level of satisfaction a person feels in regard to different life domains [9]. Recent studies based on this approach have established that satisfaction within the food and family domains are positively related to overall life satisfaction in adolescents [10,11,12,13,14,15,16,17]. Whereas satisfaction with food-related life is defined as a person’s overall assessment of their food and eating habits [18], satisfaction with family life is defined as a conscious cognitive judgment of one’s family life [19]. Various studies have addressed the relationships between emotional family support in university students; dietary restraint in adolescents; food-related parenting practices in mothers and in their adolescent children and satisfaction with food-related life, satisfaction with family life and overall life satisfaction [12,13,17].

Nevertheless, to our knowledge, no studies exist that have assessed the associations between family support, the work-life balance of both parents and their adolescent children’s satisfaction with food-related life, satisfaction with family life and overall life satisfaction. Following the recommendations of Haar et al. [20], work-life balance was conceptualized as an individual’s perception of how well his or her life roles are balanced (balance between work and the rest of life). Therefore, the objectives of the current study were (a) to explore the associations between family support and satisfaction with life, food-related life and family life among adolescent children from dual-earner families and (b) to explore the associations between both parents’ work-life balance and satisfaction with life, food-related life and family life among adolescent children in dual-earner families.

## 2. Theoretical and Empirical Background

### 2.1. Associations between Family Support, Parents’ Work-Life Balance and Adolescents’ Subjective Well-Being

Studies performed in different countries have reported that family support is related to higher levels of subjective well-being and life satisfaction [7,21,22,23,24,25,26], more satisfaction with food-related life [12,27,28,29] and greater satisfaction with family life [16,30,31] in adolescents and youth.

The relationship between the family and work domains has been widely studied in workers as negative interactions between these domains is often associated with negative outcomes for individuals, families, businesses and society, which results in family, job, marital, parental and life dissatisfaction [32,33,34,35,36]. In fact, the level of balance between work and family impacts other life domains and is likely to affect not only the individual parent but also those closest to them [33,37], such as the couple and their children [35]. However, while the impact of the family-work relationship has been extensively studied in couples for example [31,35,37,38,39,40], there remain few studies that have assessed the influence of the parental balance between work and family on their children’s well-being [5,33,41,42]. Strazdins et al. [43] found that unconventional work schedules contributed to depressive symptoms in parents, which negatively affected the well-being of their children. More recently, Lawson et al. [44] found that positive work experiences of mothers led to lower levels of negative moods after work, thus leading to higher reported levels of well-being in adolescents. Mauno et al. [41] reported that positive work-related experiences in mothers were positively and indirectly associated with children’s life satisfaction. Likewise, some studies have reported that parents’ workload is associated with conflict with their children, whereas work stress has a negative effect on the quality of family interactions [5,45,46]. Therefore, these studies suggest that the parents’ work-life balance not only affects the overall well-being of their children but also affects children’s well-being in the family domain. Another sphere that may be affected by parent work-life balance is the food domain. Some studies have reported that full-time employed mothers, as well as dual earner families with poor work-life balance, are associated with less healthful food environments [47,48,49,50,51], less encouragement for their kids to make healthy food choices [47,50] and less frequent family meals [47,50,52,53,54,55,56,57,58]. Therefore, given that some of these practices are negatively related to the level of food-related life satisfaction in adolescents [17,58,59,60], it is expected that parent work-life balance may be related to the food-related life satisfaction of their adolescent children.

Recent studies in adult samples suggest that family support is related to the balance between family and work [31,61]. Therefore, it is expected that parents that have achieved a better balance between both domains have more time to provide emotional support to their children, as suggested by Mendolia [42].

Given this background, we posed the following hypotheses:

**Hypotheses** **1 (H1).** *Perceived family support is positively associated with life satisfaction in adolescents.*

**Hypotheses** **2 (H2).** *Perceived family support is positively associated with satisfaction with family life in adolescents.*

**Hypotheses** **3 (H3).** *Perceived family support is positively associated with satisfaction with food-related life in adolescents.*

**Hypotheses** **4 (H4).** *Life satisfaction in adolescents is positively associated with (a) mothers’ work-life balance and (b) fathers’ work-life balance.*

**Hypotheses** **5 (H5).** *Satisfaction with family life in adolescents is positively associated with (a) mothers’ work-life balance and (b) fathers’ work-life balance.*

**Hypotheses 6** **(H6).** *Satisfaction with food-related life in adolescents is positively associated with (a) mothers’ work-life balance and (b) fathers’ work-life balance.*

**Hypotheses 7** **(H7).** *Perceived family support in adolescents is positively associated with (a) mothers’ work-life and (b) fathers’ work-life balance.*

However, although the level of the father’s engagement in child-rearing tasks has increased over the past few decades, mothers are still primarily responsible for raising children, thus exerting the most influence on their children’s diet [3,5,8,56,62]. It is therefore expected that the strength of the associations between adolescent life, family life and food-related life satisfaction and parents’ work-life balance will differ according to the parent’s gender.

### 2.2. Mediating Variables between Family Support, Parents’ Work-Life Balance and Adolescents’ Life Satisfaction

Feeney and Collins [63] suggested that social support exists in multiple forms and can affect behavior and well-being through several pathways. Others have suggested that certain mediators of the relationship between social support and subjective well-being explain the mechanism underlying this connection, such as satisfaction with school experience, satisfaction with family life, self-efficacy and sense of community in school [16,25,64,65,66]. More recently, Schnettler et al. [17] suggested that satisfaction with food-related life and satisfaction with family life may have mediating roles between food-related parenting practices and overall life satisfaction in adolescents.

There is increasing evidence indicating that frequent and cohesive family meals are associated with positive outcomes for adolescents and youth, such as healthier diets, higher levels of psychological well-being, food-related life satisfaction and family life satisfaction [8,9,27,28,29,58,60,67,68,69,70]. However, not all families sit down to regularly share family meals [71,72]. Some of the main barriers to having frequent family meals include conflicting work and school schedules and stressed or overworked parents [71,72]. In fact, although parents agree that eating together is important, many employed parents report that demanding work and family conditions make it difficult to do so on a daily basis [52,55,73].

Considering this information, we explored the mediating role of satisfaction with family life and food-related life through the following hypotheses:

**Hypotheses 8** **(H8).** *Perceived family support is associated with adolescents’ life satisfaction through satisfaction with family life.*

**Hypotheses 9** **(H9).** *Perceived family support is associated with adolescents’ life satisfaction through satisfaction with food-related life.*

**Hypotheses 10** **(H10).** *Mothers’ work-life balance is associated with adolescents’ life satisfaction through satisfaction with family life.*

**Hypotheses 11** **(H11).** *Fathers’ work-life balance is associated with adolescents’ life satisfaction through satisfaction with family life.*

**Hypotheses 12** **(H12).** *Mothers’ work-life balance is associated with adolescents’ life satisfaction through satisfaction with food-related life.*

**Hypotheses 13** **(H13).** *Fathers’ work-life balance is associated with adolescents’ life satisfaction through satisfaction with food-related life.*

## 3. Method

### 3.1. Sample and Procedure

A power analysis was performed using the G*power 3.1 program. Then, a minimum sample size of 272 dual-earner families with at least one adolescent child between 10 and 17 years of age in Temuco, Chile, was established (Cronbach’s alpha error prob = 0.05, effect size = 0.4, power (1-β) = 0.95, allocation ratio N2/N1 = 1.0). Through non-probability sampling, a sample of 303 dual-earner families with at least one adolescent child between 10 and 17 years of age was recruited. We collected data from more participants based on the expectation of missing data or error responses. A post hoc analysis suggested that the power (1-β) = 0.97 given Cronbach’s alpha, sample size and effect size. Table 1 shows the sociodemographic characteristics of the sample. Married and unmarried cohabiting couples were included, as a growing preference for cohabitation in lieu of legal marriage has recently been observed in Chile [74]. Participants were recruited from seven schools that serve socioeconomically diverse populations. Directors from each school signed authorization letters to conduct the research with their students and provided a list of students from fifth grade and up (corresponding to a minimum age of 10), along with their parents’ telephone numbers. Parents were contacted by trained interviewers who explained the study objectives and the strictly confidential treatment of the information obtained. Then, they provided detailed information about the questionnaires and asked if both parents and one of their children between 10 and 17 years wanted to participate in the study. Participants were given the choice to be interviewed in their homes or schools. After all parents signed written informed consent and the adolescents signed assent forms, the questionnaires were administered to both parents and one child between 10 and 17 years old by a trained interviewer. Each family member was interviewed individually without the presence of the rest of the family members. Respondent anonymity was ensured. The study was conducted between May and August 2017 and the study design was approved by the Ethics Committee of the Universidad de La Frontera (005/2016). A pilot test of the questionnaires was conducted with 20 families following the same recruitment method. As the pilot test of the instrument was satisfactory, no changes were made to the questionnaires or the interview procedure.

### 3.2. Measures

The following instruments were answered by the adolescents:

- *Satisfaction with Life Scale* (SWLS): SWLS [75] is a scale composed of five items grouped into a single dimension that is used to evaluate overall cognitive judgments about a person’s own life (e.g., “*In most ways my life is close to my ideal*”). The validated Spanish version of the SWLS was used [76,77]. Respondents were asked to indicate their degree of agreement with each statement using a 6-point Likert scale (1: completely disagree; 6: completely agree). The SWLS score corresponded to the sum of the scores from the five items.
- *Satisfaction with Food-related Life* (SWFoL): SWFoL [18] is a scale consisting of five items grouped into a single dimension that is used to evaluate a person’s overall assessment of their food and eating habits (e.g., “*Food and meals are positive elements*”). The validated Spanish version of the SWFoL was used [74,77]. Respondents were asked to indicate their degree of agreement with each statement using a 6-point Likert scale (1: completely disagree; 6: completely agree). The SWFoL score corresponded to the sum of the scores from the five items.
- *Satisfaction with Family Life* (SWFaL) scale. This scale, proposed by Zabriskie and McCormick [19], is an adaptation of the SWLS [75] in which the words “family life” replaces the word “life” in each of the five original items of the SWLS. Family satisfaction can be defined as a conscious cognitive judgment of one’s family life based on the subjective criteria of each individual [19]. The validated Spanish version of the SWFaL was used [16]. Respondents were asked to indicate their degree of agreement with each of the statements using a 6-point Likert scale (1: completely disagree; 6: completely agree). The SWFaL score corresponded to the sum of the scores from the five items.

The validated Spanish-language versions of the SWLS, SWFoL and SWFaL have shown good internal consistency in previous studies with adolescents [8,9,17,60]. The discriminant validity of the SWLS, SWFoL and SWFaL was previously demonstrated in samples of undergraduate students, adolescents and adults in Chile [8,16,17].

- *Multidimensional Scale of Perceived Social Support* (MSPSS): MSPSS [78] is a scale composed of 12 items and three subscales: significant other, family and friends. In order to measure perceived family support in this study, only the Family subscale was used. This subscale consists of four items (e.g., “*My family really tries to help me*”). The validated Spanish version of the perceived family support (PFS) subscale was used [79]. Respondents were asked to indicate their degree of agreement with each statement using a 6-point Likert scale (1: completely disagree; 6: completely agree). The PFS score corresponded to the sum of the scores from the four items.

The following instrument was answered by the mothers and fathers:

- *Work-life Balance* (WLB): WLB [80] is a scale composed of three items grouped into a single dimension (e.g., “*I manage to balance the demands of my work and personal/family life well*”). The WLB has shown good internal consistency in previous studies conducted in different countries [20]. The validated Spanish version of the WLB scale was used [81]. Respondents were asked to indicate their degree of agreement with the three statements using a 5-level Likert scale (1: completely disagree to 5: completely agree). The WLB score corresponded to the sum of the scores from the three items.

Table 2 shows the adolescents’ average SWLS, SWFoL, SWFL and PFS scores, both parents’ average WLB scores and the internal reliability (Ordinal Alpha) obtained in the present study for each scale. All scales showed good internal reliability.

Each of the three family members was then asked for their age. Mothers were asked about their number of family members and number of children. Education level and occupation of the head of the household were used to determine socioeconomic status (SES), which was classified as high and upper middle, middle-middle, lower-middle, low and very low [82].

### 3.3. Data Analysis

Descriptive analyses were conducted using SPSS v.23 (IBM, Armonk, NY, United States). To test the hypotheses, structural equation models (SEM) were conducted using MPlus 7.11. Parameters of the structural models were estimated using the Robust Unweighted Least Squares. Considering the ordinal scale of the items, the polychoric correlation matrix was used to perform the SEM analysis. Hypotheses were tested separately for each parent. In order to control for the effects of gender, age and SES of the adolescents in the model, these three variables were added with direct associations with the SWLS dependent variable.

The Tucker-Lewis Index (TLI) and the Comparative Fit Index (CFI) were used to determine the model fit of the data. The TLI and CFI indicated a good fit with values above 0.95, while 0.90 was considered a cut-off point for establishing an acceptable fit. In addition, the Root Mean Square Error of Approximation (RMSEA) was considered. The RMSEA is a poorness of fit measurement. A good fit is found when the value of the RMSEA is lower than 0.06, whereas an acceptable fit corresponds to a value lower than 0.08 [83,84].

Following Lau and Cheung [85], we tested the mediating roles of satisfaction with family life and satisfaction with food-related life via structural equation model through a bias-corrected (BC) bootstrap confidence interval using 1000 samples. The mediating role was established when the BC confidence interval for the mediation effect did not include zero.

## 4. Results

**Figure 1:**
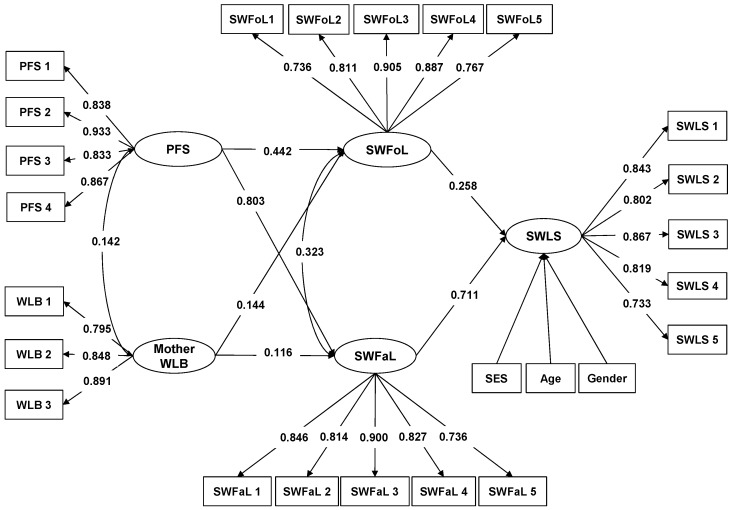
Path diagram of the model that explains the relationships between Perceived Family Support (PFS), mother’s Work-life Balance (Mother WLB) and Satisfaction with Family Life (SWFaL) and Satisfaction with Food-related Life (SWFoL) and between SWFoL, SWFaL and Satisfaction with Life Scale (SWLS) in adolescents. SES: Socioeconomic status. Only significant path coefficients are shown (*p* < 0.05). The items of each scale are shown in the Appendix A.

**Figure 2:**
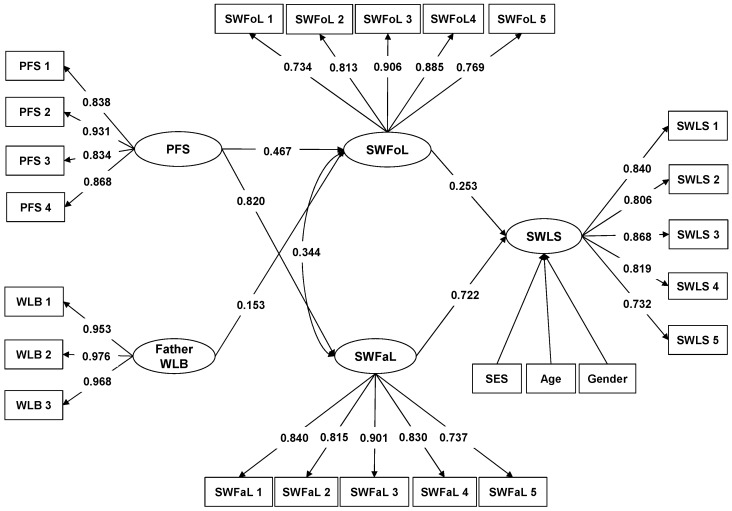
Path diagram of the model that explains the relationships between Perceived Family Support (PFS), fathers’ Work-life Balance (Father WLB) and Satisfaction with Family Life (SWFaL) and Satisfaction with Food-related Life (SWFoL) and between SWFoL, SWFaL and Satisfaction with Life Scale (SWLS) in adolescents. SES: Socioeconomic status. Only significant path coefficients are shown (*p* < 0.05). The items of each scale are shown in the Appendix A.

Considering that the hypotheses were tested separately for each parent, Figure 1 shows the results of the structural model that considered the mothers’ WLB while Figure 2 shows the results of the structural model that considered the fathers’ WLB.

### 4.1. Testing Relationships with Structural Equation Modelling

Having controlled for adolescents’ gender, age and SES, the structural model that considered the mothers’ WLB (Figure 1, CFI = 0.990; TLI = 0.988; RMSEA = 0.022), as well as the structural model that considered the fathers’ WLB (Figure 2, CFI = 0.990; TLI = 0.980; RMSEA = 0.021), had fit indices that showed good fit with the data. In both models, the path coefficient between SWFoL and SWLS was positive, confirming that food-related life satisfaction is positively associated with overall life satisfaction in adolescents. Similarly, the path coefficient between SWFaL and SWLS was also positive, supporting the finding that satisfaction with family life is positively associated with overall life satisfaction in adolescents. The positive and significant correlation between SWFoL and SWFaL confirmed that food-related life satisfaction was positively associated with family life satisfaction in adolescents.

In both models (Figure 1 and Figure 2), the path coefficients between PFS and SWLS were not significant and thus did not support the hypothesis that perceived family support is positively associated with life satisfaction in adolescents (H1). The path coefficients between PFS and SWFaL were positive and significant in both models, supporting that perceived family support is positively associated with satisfaction with family life in adolescents (H2). Similarly, the path coefficients in both models between PFS and SWFoL were positive and significant, meaning that perceived family support is positively associated with satisfaction with food-related life in adolescents (H3).

The path coefficients between mothers’ WLB and SWLS (Figure 1) and between fathers’ WLB and SWLS (Figure 2) were not significant. Thus, they did not support the hypotheses that mothers’ work-life balance is positively associated with life satisfaction in adolescents (H4a) and that fathers’ work-life balance is positively associated with life satisfaction in adolescents (H4b). The path coefficient between mothers’ WLB and SWFaL was positive and significant (Figure 1), supporting the hypothesis that mothers’ work-life balance is positively associated with satisfaction with family life in adolescents (H5a). However, the non-significant path coefficient between fathers’ WLB and SWFaL (Figure 2) did not support the expectation that fathers’ work-life balance is positively associated with satisfaction with family life in adolescents (H5b). The path coefficient between mothers’ WLB and SWFoL was positive and significant (Figure 1), meaning that mothers’ work-life balance is positively associated with satisfaction with food-related life in adolescents (H6a). Similarly, the path coefficient between fathers’ WLB and SWFoL was positive and significant (Figure 2), supporting the hypothesis that fathers’ work-life balance is positively associated with satisfaction with food-related life in adolescents (H6b).

The positive and significant correlation between mothers’ WLB and PFS (Figure 1) supports the hypothesis that mothers’ work-life balance is positively associated with perceived family support in adolescents (H7a). The non-significant correlation between father’s WLB and PFS (Figure 2) did not support the expectation that fathers’ work-life balance is positively associated with perceived family support in adolescents (H7b).

In summary, the main differences between the structural model that considered the mothers’ WLB (Figure 1) and the structural model that considered the fathers’ WLB (Figure 2) were the lack of significant associations between the fathers’ WLB and the adolescents’ satisfaction with family life, as well as between the fathers’ WLB and adolescents’ perceived family support.

### 4.2. Testing Mediating Roles of Satisfaction with Family and Food-Related Life

SWFaL has a mediating role between PFS and adolescents’ SWLS (H8), since the 95% BC confidence interval does not include zero in the model that considers the mothers’ WLB (Table 3) or in the model that considers the fathers’ WLB (Table 4). This indicates that the mediating roles are significantly different from zero. SWFoL also has a mediating role between PFS and adolescents’ SWLS (H9), since the 95% BC confidence interval does not contain zero in the model that considers the mothers’ WLB (Table 3) or in the model that considers the fathers’ WLB (Table 4), indicating that the mediating role is significantly different from zero. The mediating roles from SWFaL and SWFoL were complete, since the direct relationships between PFS and the adolescents’ SWLS were statistically significant in the absence of the mediator but these relationships became statistically non different from zero in the presence of the mediator (Table 5 and Table 6).

Similarly, SWFaL has a mediating role between the mothers’ WLB and the adolescents’ SWLS (H10) (Table 3), which was complete (Table 5). SWFaL has no mediating role between the fathers’ WLB and the adolescents’ SWLS (H11), since the 95% BC confidence interval does include zero (Table 4).

SWFoL has a mediating role between the mothers’ WLB and the adolescents’ SWLS (H12) (Table 3), which was also complete (Table 5). SWFoL also has a mediating role between the fathers’ WLB and the adolescents’ SWLS (H13) (Table 4), which was also complete (Table 6).

Finally, given the lack of a significant correlation between PFS and WLB, the lack of a significant path coefficient between WLB and SWFaL and the lack of a mediating role of SWFaL between WLB and SWLS in the model that considered the father’s WLB, the gender of the parents appeared to play a moderating role.

## 5. Discussion

This study provides new insights regarding the associations between life satisfaction, food-related life satisfaction and family life satisfaction, including the associations between adolescents’ perception of family support and the work-life balance of both parents in dual-earner families in a developing country.

Regarding the first objective, perceived family support was not found to be directly associated with the adolescents’ overall life satisfaction, which was contrary to was expected [7,21,22,23,24,25,26]. However, our results show that life satisfaction among adolescents was indirectly influenced by perceived family support through satisfaction with family life and satisfaction with food-related life. Thus, our findings are consistent with previous research reporting on the existence of mediators in the association between social support and subjective well-being in adolescents and youth [25,64,65,66]. In part, our results are also consistent with a recent study reporting on satisfaction with family life as a mediator between family support and satisfaction with life in undergraduate students [16]. However, the present study also makes a significant contribution by showing that satisfaction with food-related life may be another mediator of this association. These results suggest that adolescents with high levels of perceived family support are likely to experience higher levels of satisfaction with family and food-related life, which results in high levels of life satisfaction. This is consistent with the bottom-up theoretical approaches to life satisfaction [9]. The positive association between perceived family support and adolescent food-related life satisfaction may be explained by the affective dimension of meals as a moment of family unity, since family meals have been identified as the moment when parents offer emotional support to their children, thus improving the satisfaction with food-related life in adolescents [27,29,86,87]. Nevertheless, according to Cohen [88], the association between perceived family support and satisfaction with food-related life was of medium strength, whereas the relationship between perceived family support and satisfaction with family life was of high strength. Therefore, it is possible to suggest that family relationships are a strong predictor of adolescent subjective well-being in the family domain. Nevertheless, as our findings show, family support should be encouraged in dual-earner families with adolescent children in order to improve both adolescents’ family life and food-related life satisfaction, as both indirectly lead to higher life satisfaction.

Regarding our second objective, the work-life balance of both parents was not related to their adolescent children’s life satisfaction, contrary to was expected [41,43,44]. However, our results are consistent with studies that show no relationship between a mother’s employment and her children’s well-being [42,89]. Wills and Brauer [89] attributed this lack of association to the fact that children have adapted to their mothers’ work outside the home. Taking this into account and considering the traditional role of fathers as the primary breadwinner [48], it is not surprising that father’s work-life balance was not related to their adolescent children’s life satisfaction. In this regard, Kinkead et al. [5] reported that adolescent children do not dramatically perceive the tensions and conflicts related to their parents’ relationship between family and work, which is likely due to the recognition of their efforts on behalf of family well-being, despite them being absent for long periods of time.

Regardless, our results show that satisfaction with family life serves a complete mediating role between mother’s work-life balance and their adolescent children’s life satisfaction, though this was not the case in work-life balance among fathers. Although the lack of association between father’s work-life balance and their adolescent children’s family life satisfaction merits further research, one possible explanation may be related to the primacy of the mother-child relationship [90]. In this regard, although mothers work outside the home, they continue to bear the primary responsibility for raising children [3] and are still expected to take on more responsibilities to keep their work investment from intruding into family life [35]. Accordingly, there is evidence that mothers are more prone to adapt their working hours to fit around their families than fathers, which allows mothers to promote a positive mother-child relationship [5,41,45,46,56,73]. In addition, mothers may be more sensitive to how their work affects their children’s eating habits, adapting their work schedules to the feeding schedules of their children, which may also be related to higher levels of family life satisfaction [54,56,73]. In this regard, Schnettler et al. [60] found that adolescent children who have frequent meals with their mothers were more satisfied with their family life, whereas Jacob et al. [91] found that working mothers who have the time to have family meals have better relationships with their children. Therefore, special emphasis should be placed on developing intervention strategies that improve a mother’s work-life balance in order to improve her adolescent children’s satisfaction with family life, which in turn improves their overall life satisfaction.

Our findings also show that satisfaction with food-related life has a complete mediating role between both parents’ work-life balance and their adolescent children’s life satisfaction. This is a notable result that reflects the increasing participation of fathers in childrearing, specifically in regard to family meals, due to women’s growing participation in the workforce [56]. Indeed, there is evidence showing that, in dual-earner families, fathers want a more balanced relationship between work and family, which would allow them to participate in regular family meals [48]. These results may be related to the fact that parents who experience higher levels of work-life balance have more frequent family meals [47,48,52,54,56,73], which are associated with higher levels of food-related life satisfaction in adolescents, due since sharing meals is a good time for communication and expression of affection between parents and their children [8,27,28,29,60]. Therefore, the work-life balance of both parents should be promoted in order to improve their adolescent children’s well-being in the food domain, which indirectly promotes better levels of life satisfaction.

Regarding the relationship between the work-life balance of both parents and adolescents’ perception of family support, our findings confirmed this association in work-life balance among mothers but not among fathers. This suggests that women are still reserving time for their children despite competing demands [56]. In addition, although the level of fathers’ engagement in childrearing tasks has increased over the last few decades, our findings suggest that their greater participation may not have an impact in their emotional relationships with their children [56]. In this regard, Kinkead et al. [5] found that adolescent children perceive their mothers as having a more important participation in matters related to emotional care and expressions of affection than their fathers. Nevertheless, further research is required in order to better understand why the fathers’ involvement does not translate into an improvement of positive aspects, such as greater emotional support and higher levels of satisfaction with family life in their adolescent children.

It should also be noted that the association between mothers’ work-life balance and their children’s food-related and family life satisfaction, as well as the association between fathers’ work-life balance and their children’s food-related life satisfaction, were of low strength. In fact, they were of much lower strength than the associations between perceived family support and the adolescents’ satisfaction with family life (high strength) and satisfaction with food-related life (medium strength). In addition, although mothers’ work-life balance was positively associated to their adolescent children’s perceived family support, this association was also of low strength [88]. Therefore, although parents are generally perceived as a key source of support by their adolescent children [21], it is possible to hypothesize that other family members, such as siblings and/or grandparents, are providing some of the family support that children need during adolescence in dual-earner families. Nevertheless, it is also possible to hypothesize that parents give support to their adolescent children in a way that is independent from their level of work-life balance. Therefore, further research is required in order to better understand the family-related variables that allow children in dual-earner households to receive support from their families.

## 6. Limitations and Conclusions

One of the limitations of this study is its cross-sectional design, forcing us to be cautious about causal relationships. Thus, in order to test causality, future studies are required that consider longitudinal designs. Another limitation is related to the non-probabilistic nature of the sample and its relatively small size, as well as the fact that it examined families from only one city in one country, which limits the generalization of our results. Therefore, cross-cultural studies are needed, specifically those that compare the analyzed associations to other developing countries and to developed countries from different geographic areas, given that cultural variables may affect the influence of gender roles and the level of work-life balance [92].

Furthermore, all data were self-reported. Thus, responses may have been affected by social desirability. Part of this paper’s discussion related higher levels of satisfaction with food-related and family life with more frequent family meals even though participants were not asked about the frequency of family meals. Therefore, future studies are required to corroborate these associations. In addition, since the questionnaire did not distinguish between meals, it is possible that some adolescents answered the SWFoL thinking about dinner (the main family meal in Chile), whereas others may have answered thinking about lunch (which is mainly eaten by adolescents during school). This may explain the lower strength of the association between adolescents’ perceived family support and satisfaction with food-related life. Therefore, future research should explore the level of satisfaction with food-related life regarding different meals throughout the day. Also, we did not ask about the parents’ type of employment nor the number of working hours. Therefore, it was not possible to associate parents’ work-life balance to their job conditions. These variables should be taken into account in future studies, given that they may moderate the levels of work-life balance in working parents [33,36,42]. Finally, we did not assess family diet quality or food-related parenting practices used by parents, which may differ according to the level of the parents’ work-life balance [47,48,49,50,51]. Future studies should incorporate these variables, since they may affect the nutritional status and well-being in adolescents [9,13,17,58,60,67,68]. Therefore, in addition to the adolescents’ age, gender and socioeconomic status, future studies may also control for adolescents’ body mass index.

However, despite these limitations, this is the first study that explored the associations between family support, the work-life balance of both parents, satisfaction with life, food-related life and family life among adolescent children from dual-earner families. Our findings showed a high positive association between family support and satisfaction with family life, as well as a medium positive association between family support and satisfaction with food-related life. In addition, we found that satisfaction with family life and satisfaction with food-related life had complete mediating roles between family support and adolescent life satisfaction, which contributes to the existing literature regarding mediators of the relationship between social support and subjective well-being. Therefore, familial support should be promoted in order to improve adolescents’ family life and food-related life satisfaction, which would positively impact adolescents’ life satisfaction in dual-earner families. This is especially relevant in adolescents with mental health problems such as depression or anxiety, given that family support predicts the decline of these mental health symptoms during the adolescence [93]. Our findings also showed positive associations between the mothers’ work-life balance and adolescents’ family life and food-related life satisfaction, although these associations were of low strength. Conversely, only a low strength positive association was found between fathers’ work-life balance and adolescents’ satisfaction with food-related life. In addition, we found that satisfaction with family life had a complete mediating role between mothers’ work-life balance and their adolescent children’s life satisfaction, whereas satisfaction with food-related life had a complete mediating role between the work-life balance of both parents and their adolescent children’s life satisfaction.

Considering that mothers’ work-life balance was also related to their adolescent children’s perception of family support, special emphasis should be placed on developing policies and strategies aimed at improving mothers’ work-life balance in order to enhance adolescent satisfaction in different domains of life in dual-earner families. This is especially relevant in Chile given that 900,000 additional women are expected to join the country’s workforce in the medium-term. Future studies should also assess the relationship between satisfaction with life, food-related life and family life among adolescent children and other family-related variables, such as parents’ mental health and family functioning.

## Figures and Tables

**Table 1:** Sample characteristics (n = 303).

Characteristic	Total Sample
Mother’s age [Mean (*SD*)]	40.9 (7.4)
Father’s age [Mean (*SD*)]	43.2 (7.2)
Number of family members [Mean (*SD*)]	4.3 (1.1)
Number of children [Mean (*SD*)]	2.4 (1.0)
Interviewed children’s age [Mean (*SD*)]	13.3 (2.4)
Interviewed children’s gender (%)	
Female	51.5
Male	48.5
Socioeconomic status (%)	
High and upper-middle	11.2
Middle-Middle	20.8
Lower-Middle	37.0
Low	21.5
Very low	9.6

**Table 2:** Mean, standard deviation (*SD*) and internal reliability (Ordinal Alpha) of the Satisfaction with Life Scale (SWLS), Satisfaction with Food-related Life (SWFoL), Satisfaction with Family Life (SWFaL) Perceived Family Support (PFS) and Work-life Balance (WLB) scales.

Sample/Scale	Mean (*SD*)	Ordinal Alpha
Adolescent SWLS	25.5 (4.1)	Total sample = 0.902, male = 0.910, female = 0.910
Adolescent SWFoL	24.4 (4.7)	Total sample = 0.891, male = 0.920, female = 0.910
Adolescent SWFaL	25.7 (4.5)	Total sample = 0.909, male = 0.910, female = 0.920
Adolescent PFS	21.3 (3.5)	Total sample = 0.920, male = 0.910, female = 0.950
Work-life Balance		
Mother	12.5 (2.2)	0.876
Father	12.1 (2.3)	0.919

**Table 3:** Bias-Corrected confidence intervals of specific mediation effects in the model that includes the mothers’ work-life balance (Mother WLB).

Effects	Lower 2.5%	Estimate	Upper 2.5%
From PFS to SWLS Specific Indirect			
SWLS			
SWFaL			
PFS	0.425	0.571	0.717
SWLS			
SWFoL			
PFS	0.059	0.114	0.169
From mother WLB to SWLS Specific Indirect			
SWLS			
SWFaL			
Mother WLB	0.017	0.082	0.147
SWLS			
SWFoL			
Mother WLB	0.003	0.037	0.071

PFS: Perceived family support. SWLS: Satisfaction with Life. SWFaL: Satisfaction with Family Life. SWFoL: Satisfaction with Food-related life.

**Table 4:** Bias-Corrected confidence intervals of specific mediation effects in the model that includes the fathers’ work-life balance (Father WLB).

Effects	Lower 2.5%	Estimate	Upper 2.5%
From PFS to SWLS Specific Indirect			
SWLS			
SWFaL			
PFS	0.449	0.592	0.735
SWLS			
SWFoL			
PFS	0.062	0.118	0.175
From father WLB to SWLS Specific Indirect			
SWLS			
SWFaL			
Father WLB	−0.048	0.014	0.077
SWLS			
SWFoL			
Father WLB	0.009	0.039	0.068

PFS: Perceived family support. SWLS: Satisfaction with Life. SWFaL: Satisfaction with Family Life. SWFoL: Satisfaction with Food-related life.

**Table 5:** Confidence intervals in the model that includes the mothers’ work-life balance (Mother WLB).

Effects	Lower 2.5%	Estimate	Upper 2.5%
SWLS on			
PFS	−0.177	0.007	0.158
Mother WLB	−0.038	0.033	0.111

PFS: Perceived family support. SWLS: Satisfaction with Life.

**Table 6:** Confidence intervals in the model that includes the fathers’ work-life balance (Father WLB).

Effects	Lower 2.5%	Estimate	Upper 2.5%
SWLS on			
PFS	−0.179	0.005	0.155
Father WLB	−0.024	−0.034	0.088

PFS: Perceived family support. SWLS: Satisfaction with Life.

## Appendix A

Items of the Satisfaction with Life Scale (SWLS), Satisfaction with Food-related Life (SWFoL), Satisfaction with Family Life (SWFaL), Perceived Family Support (PFS) and Work-life Balance (WLB) scales:

SWLS 1	In most ways my life is close to my ideal
SWLS 2	The conditions of my life are excellent
SWLS 3	I am satisfied with my life
SWLS 4	So far I have gotten the important things I want in life
SWLS 5	If I could live my life over, I would change almost nothing.
SWFoL 1	Food and meals are positive elements
SWFoL 2	I am generally pleased with my food
SWFoL 3	My life in relation to food and meals is close to ideal
SWFoL 4	With regard to food, the conditions of my life are excellent
SWFoL 5	Food and meals give me satisfaction in daily life.
SWFaL 1	In most ways my family life is close to my ideal
SWFaL 2	The conditions of my family life are excellent
SWFaL 3	I am satisfied with my family life
SWFaL 4	So far I have gotten the important things I want in family life
SWFaL 5	If I could live my family life over, I would change almost nothing
PFS 1	My family really tries to help me
PFS 2	I get the emotional help and support I need from my family
PFS 3	I can talk about my problems with my family
PFS 4	My family is willing to help me make decisions
WLB 1	I manage to balance the demands of my work and personal/family life well
WLB 2	Nowadays, I seem to enjoy every part of my life equally well
WLB 3	I manage to balance the demands of my work and personal/family life well
